# Static and dynamic formant scaling conveys body size and aggression

**DOI:** 10.1098/rsos.211496

**Published:** 2022-01-12

**Authors:** Andrey Anikin, Katarzyna Pisanski, David Reby

**Affiliations:** ^1^ Division of Cognitive Science, Lund University, Lund, Sweden; ^2^ ENES Sensory Neuro-Ethology lab, CRNL, Jean Monnet University of Saint Étienne, UMR 5293, 42023, St-Étienne, France

**Keywords:** vocal tract length, body size, formants, acoustic communication, dynamic

## Abstract

When producing intimidating aggressive vocalizations, humans and other animals often extend their vocal tracts to lower their voice resonance frequencies (formants) and thus sound big. Is acoustic size exaggeration more effective when the vocal tract is extended before, or during, the vocalization, and how do listeners interpret within-call changes in apparent vocal tract length? We compared perceptual effects of static and dynamic formant scaling in aggressive human speech and nonverbal vocalizations. Acoustic manipulations corresponded to elongating or shortening the vocal tract either around (Experiment 1) or from (Experiment 2) its resting position. Gradual formant scaling that preserved average frequencies conveyed the impression of smaller size and greater aggression, regardless of the direction of change. Vocal tract shortening from the original length conveyed smaller size and less aggression, whereas vocal tract elongation conveyed larger size and more aggression, and these effects were stronger for static than for dynamic scaling. Listeners familiarized with the speaker's natural voice were less often ‘fooled’ by formant manipulations when judging speaker size, but paid more attention to formants when judging aggressive intent. Thus, within-call vocal tract scaling conveys emotion, but a better way to sound large and intimidating is to keep the vocal tract consistently extended.

## 1. Introduction

Looking and sounding impressively large is often advantageous for group-living animals, particularly during dominance displays in males, leading to anatomical and behavioural adaptations for advertising—and often exaggerating—body size [1,2]. It is now well established that vocal tract elongation is an effective method of acoustic size exaggeration in humans [3–5] and numerous other species of mammals [6]. However, little is known about *dynamic* aspects of vocal tract adjustments. Experimental manipulations that mimic the acoustic consequences of extending or shortening the vocal tract tend to present listeners with only the end product of statically long or short vocal tracts [3,4,7]. By contrast, in real animal and human vocalizations, vocal tract length (VTL) can be modified gradually during vocalizing. We investigated the potential communicative significance of this dynamic aspect of VTL changes.

The region between the source of acoustic excitation—in mammals, laryngeal vocal folds—and the point at which the sound escapes through the lips or nostrils acts as a resonator. The length of this resonator is roughly inversely proportional to the frequencies of resonances, also known as formants [8]. Therefore, listeners can estimate the size of the vocal tract from the frequency bands that it amplifies. Of course, knowing the VTL is not the same as knowing the body size of a caller; in fact, formant frequencies explain less than 10% of the variance in actual height within human adults of either sex [9–11]. Furthermore, humans [12,13] and many other animals [6] can vary the degree of mouth opening, protrude the lips or pull the larynx up and down, varying their effective VTL [8]. However, there are anatomical constraints on the scope of VTL adjustments within each species [14], so formants provide more reliable information about body size than does voice pitch [1,5], which can vary widely in the absence of hard anatomical constraints on the length of vocal folds and therefore shows little correlation with actual size, particularly within age–sex classes [11]. Human listeners do use formant frequencies to estimate the speaker's size: raising all formant frequencies by the same proportion, which is tantamount to a uniform scaling of the vocal tract, makes the speaker appear smaller, while lowering all formants increases the speaker's apparent body size [3,4,7,15].

When a vocal cue is perceptually associated with physical size, it can also be used to convey other messages that can be mapped onto a ‘size code’, such as dominance or aggression. For example, humans and many other animals possess a strong sensory bias that leads them to associate low auditory frequencies with large size, which may help to explain why vocalizers tend to drop their voice pitch in aggressive vocalizations [16,17]. Similarly, there are also some reports that formant frequencies affect perceived emotion, with lower formants signalling anger [12,18]. On the production side, a noticeable increase in average VTL was reported for angry and sad compared to happy speech in a recent imaging study [12]. This suggests that vocalizers capable of changing their VTL may do so not merely to sound large, but to express a range of emotions and intentions.

A largely untested aspect of VTL as a vocal cue is whether perceiving that formants are high or low on average (static cue) is equivalent to hearing the vocal tract being extended or shortened within one vocalization or utterance (dynamic cue). In the case of voice pitch, for example, the speaker-average pitch is consistently (and often erroneously) associated with size [11], while its dynamic component—intonation—expresses a wide range of distinct meanings [17,19]. In particular, an apparent effort on the speaker's part to deviate from habitual voice characteristics is often interpreted by listeners as a sign of a particular motivation such as sexual interest [20] or anger [21]. Similarly to intonation, dynamic changes in VTL may also convey specific information to listeners. Smiling speech can be considered a type of dynamic VTL control because spreading the lips shortens the vocal tract and temporarily raises formant frequencies [22], and the resulting ‘auditory smile’ is indeed perceived by listeners as an expression of happiness [23].

In perhaps the most direct experimental test of dynamic VTL manipulations in humans, Chuenwattanapranithi *et al*. [15] investigated the effect of dynamically lowering or raising the larynx in isolated vowels created with an articulatory speech synthesizer. Static formant manipulations affected the speaker's apparent size, while dynamic manipulations affected only the perceived emotion conveyed by the vocalization: falling formants conveyed anger, while rising formants conveyed happiness. The authors speculated that changes in VTL might communicate an effort to exaggerate or extenuate apparent size, which listeners in turn interpret as an expression of intent or emotion. However, this pioneering study employed short isolated vowels, which have limited ecological validity, and the results depended on the tested vowel. Furthermore, the manipulations were considerably more complicated than a simple scaling of the vocal tract, because the authors attempted to preserve vowel quality while emulating articulatory movements such as lowering or raising the larynx or protruding the lips.

**Figure 1.**
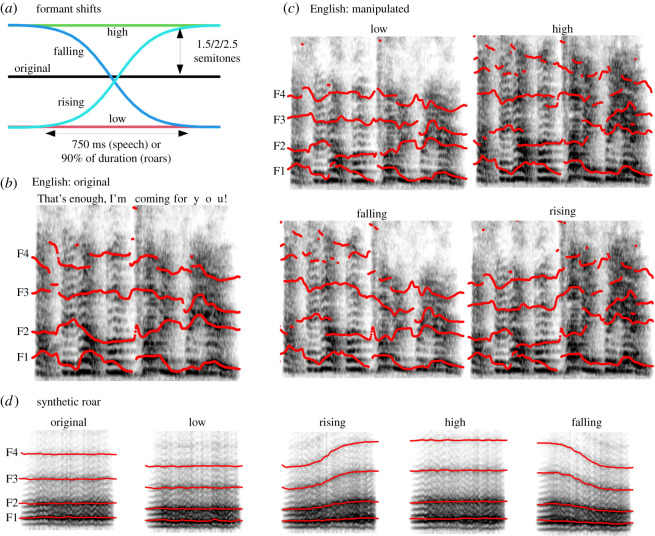
An illustration of formant manipulations in Experiment 1. (*a*) A spectrogram of the original English utterance with the approximate contours of the first four formants traced with red lines. Notice the downward trajectory of formants F3 and F4, suggesting some natural vocal tract elongation in the original, unmanipulated recording. (*b*) Formant shifts per condition, relative to the original formant frequencies. (*c*) The same utterance with formants shifted by 2.5 semitones, or approximately 15.5%. (*d*) Spectrograms of a synthetic roar with formants shifted by 2.5 semitones. Notice the flat original formant contours and clean S-curve transitions in the roar. All spectrograms have a frequency range of 0 to 5 kHz.

In our two perceptual experiments, we measured the effect of static and dynamic VTL manipulations on perceived body size and emotional state (aggression and emotion intensity) in aggressive speech and nonverbal vocalizations. Five conditions were compared in Experiment 1: the original utterance, formants experimentally manipulated to be statically high or low relative to the original value, and formants dynamically raised or lowered around the original value (figure 1*b*). Because the average formant frequencies, and therefore the average apparent VTL, were not affected by dynamic manipulations, this design can be seen as a model of VTL changing around its neutral value, which enabled us to distinguish between the average apparent VTL over time and its direction of change (elongation or shortening). To complement this controlled manipulation with a more ecologically valid scenario, in Experiment 2, we presented a different sample of listeners with angry utterances in which formant frequencies were experimentally scaled statically or dynamically from the same neutral value. This design enabled us to test whether size and aggression could be communicated more effectively by extending the vocal tract before vocalizing and keeping it consistently extended throughout a call or, instead, by letting the listeners hear the gradual process of vocal tract elongation.

## 2. Experiment 1

We tested the effect of formant manipulations in aggressive speech and in aggressive synthetic roars. Human roar-like aggressive nonverbal vocalizations provide an excellent model for studying the effect of VTL manipulations: they are ecologically valid, largely homologous to animal calls in their form and function, naturally affect-ridden, yet free from phonemic constraints and linguistic content. They have also been shown to communicate various emotional states, such as anger and pain [24], as well as body size and physical strength [25]. Speech has the drawback that rapid articulatory movements and natural VTL dynamics may mask experimental formant manipulations: for example, a speaker may already be gradually extending their vocal tract before any formant manipulations are applied. On the other hand, speech perception depends crucially on tracking individual formant frequencies with an in-built mechanism for vocal tract normalization because the same frequencies of the first two formants may correspond to different phonemes depending on the size and shape of the vocal tract [3,7,26]. As a result, human listeners are exquisitely sensitive to small VTL manipulations in speech: for example, just noticeable differences for speaker size are 30–70% smaller for syllables than for isolated vowels [27]. To account for the possibility that the effectiveness of VTL estimation may be less pronounced in an unfamiliar language, and to make our results more generalizable, we included both a familiar (English) and an unfamiliar (Persian) language.

The main outcomes of interest were perceived speaker height and aggression. Ohala's frequency code [17] predicts that gradually lowering formant frequencies should convey large size and an aggressive attitude, while raising them should have the opposite effect, as also reported by Chuenwattanapranithi *et al*. [15]. To distinguish between aggression and general arousal, we added an additional response scale for emotion intensity; perceived authenticity was also measured to check whether experimental manipulations sounded natural.

### 2.1. Methods

#### 2.1.1. Stimuli

Samples of aggressive English speech (*n* = 39) were taken from a corpus of recordings of British drama students, who were instructed to imagine that they were about to attack someone in a fight and to yell ‘That's enough, I'm coming for you!’ [25]. Recordings of aggressive Persian speech (*n* = 43) were obtained from ShEMO—an open corpus of emotional speech compiled from radio plays [28]. In contrast with the lexically identical English recordings, the Persian utterances were taken from different contexts and were not repetitions of the same phrase. For both English and Persian, we selected a single recording from each speaker, aiming to keep the duration roughly between 1 and 2 s. Long pauses between words were removed because otherwise the manipulated formant transitions in the middle of the utterance would be difficult to hear. We avoided recordings with a very high fundamental frequency (above approximately 400–500 Hz) because the source and filter had to be clearly separable for formant shifting to work without affecting pitch. The recordings were downsampled to 16 000 Hz and high-pass filtered over 50 Hz to prevent high- and low-frequency noise from introducing artefacts during resynthesis, then normalized for peak amplitude.

Nonverbal angry vocalizations are often high-pitched and noisy, with broad or poorly defined formants, whose manipulations turned out to be difficult to hear. For this reason, and because we wanted to avoid VTL fluctuations in unmanipulated stimuli, we opted to create fully synthetic roars based on recordings of natural human roars from two published corpora [24,25]. The synthesis was performed with *soundgen* following the procedure previously validated in the context of synthesizing human nonverbal vocalizations, including roars [29]. Pitch contours and average formant frequencies were extracted from the original recordings, but the formant structure could be fully controlled, enabling us to enhance the prominence and decrease the bandwidth of formants and making formant manipulations more salient. Crucially, roars in the *original* condition were synthesized with perfectly flat formant tracks—that is, without any change in vowel quality or apparent VTL. As a result, formant transitions in the rising and falling conditions followed exactly the S-curves in figure 1*a*, while in speech they were superimposed on the underlying changes caused by articulatory movements (figure 1*b*,*c*). Roars were tested in a pilot study, and those with low aggression ratings were excluded or modified to sound more aggressive. We included 38 roar prototypes in the main experiment.

#### 2.1.2. Manipulations

Roars were synthesized directly with the desired formant contours, while the recordings of English and Persian angry utterances were modified with a phase vocoder implemented in the *shiftFormants()* function in *soundgen* [29], which uniformly scaled all formants without affecting the fundamental frequency. The nature of formant manipulations per condition was as follows (figure 1*a*):

— *original* = no change;
— *low*/*high* = all formant frequencies were scaled down (*low*) or up (*high*) relative to the original by the same musical interval (1.5, 2 or 2.5 semitones, equivalent to approximately 9%, 12% and 15.5%) over the entire recording;
— *rising* = all formant frequencies started at the level of the *low* condition, then rose (over 750 ms in speech and 90% of the call's duration in roars) in an S-curve to the level of the *high* condition, crossing the original level in the middle of the recording;
— *falling* = the reverse of *rising*: all formant frequencies started at the level of the *high* condition and descended to that of the *low* condition in a logistic S-curve.

Because formant shifts in the *rising* and *falling* condition were symmetric about the midpoint of the utterance, the mean formant frequencies and thus apparent VTL were exactly the same as in the original on a logarithmic (musical) scale. The shape and duration of formant transitions were chosen to sound natural: 750 ms worked well for speech samples (mean duration 1.7 s, range [1.3, 2.0] for English; 1.6 s, [1.0, 2.2] for Persian), but many roars were shorter (mean 0.9 s, range [0.5, 1.4]), so we applied the formant shift over 90% of the roars' duration. A pilot experiment with transitions over 500 ms and 1000 ms (400 clips of angry Persian speech, 59 listeners) failed to reveal a clear effect of transition duration. The magnitude of formant shifts was chosen so as to be clearly audible, but not unnaturally strong. For example, a shift of 2 semitones corresponds to a shortening or lengthening of a 15 cm-long vocal tract by 1.8 cm, which is within the range of naturally observed VTL changes [12,13] and well above the previously reported just noticeable differences of 4–9% for formant shifts and speaker size [4,7,27]. Transitions were logistic S-curves defined on a logarithmic (musical) scale, ensuring that the average log-frequency of each formant was not affected by the manipulation.

With five conditions and three manipulation strengths for each of 120 (39 English + 43 Persian + 38 roars) prototypes, we created a total of 1800 (120 × 15) vocal stimuli. All audio stimuli and scripts for their creation are available at http://cogsci.se/publications.html.

#### 2.1.3. Procedure

English speech, Persian speech and roars were tested on independent samples of participants, but otherwise the procedure was identical. We obtained listeners’ explicit ratings of the manipulated stimuli on four response scales in an online experiment. Each participant rated as many stimuli as there were prototypes (39, 43 or 38) in two blocks, each with a randomly selected response scale (height, aggression, emotion intensity or authenticity). The order of blocks and trials within blocks was randomized for each participant, under the constraint that the same stimulus prototype should never occur twice in the same block. All four rating scales were horizontal visual analogue scales labelled at the extremes, without tick marks but with grey stripes. The meaning of each response scale was clarified with short vignettes, as follows:

— *Height*. How tall does this person sound? If you were to describe the person based on their voice, how tall would you guess they are?
— <Extremely short> to <Extremely tall>
— *Aggression*. How aggressive does this person sound? Please indicate whether the sound conveys aggression (anger or even an intention to attack).
— <Not at all aggressive> to <Extremely aggressive>
— *Emotion intensity*. How agitated/excited does this person sound? Please indicate whether the person sounds calm or agitated/excited.
— <Completely calm> to <Extremely agitated>
— *Authenticity*. How authentic/realistic do you find this vocalization? Does this sound realistic or natural, as something you might hear in real life?
— <Not at all authentic> to <Extremely authentic>

#### 2.1.4. Participants

All participants were recruited on the online testing platform Prolific (https://www.prolific.co/) and compensated for their time. All self-reported to have normal hearing and to be fluent in English, but unfamiliar with Persian. Out of the total of 607 included participants, 61% self-identified as male and 39% as female; the mean age was 24 ± 7 years (range 18 to 62). Sample sizes were chosen to ensure sufficient precision of estimates of population-level effect sizes in Bayesian multilevel models. This precision depends on the number of prototypes, the number of times each stimulus is rated on each scale, and the consistency of ratings across participants and prototypes. Thus, with a sample size of 607, each of 1800 experimental stimuli was rated on average 6.7 times on each of the four response scales, which provided sufficient precision on the estimates of manipulation effects at the population level, namely an average 95% CI width of 3.5% at the lowest level of analysis (manipulation effects for individual experiments and manipulation strengths) and only 2% when averaging across English/Persian/roars. This is ample for detecting and accurately describing all substantively non-trivial effects of formant manipulations.

#### 2.1.5. Data analysis

**Figure 2.**
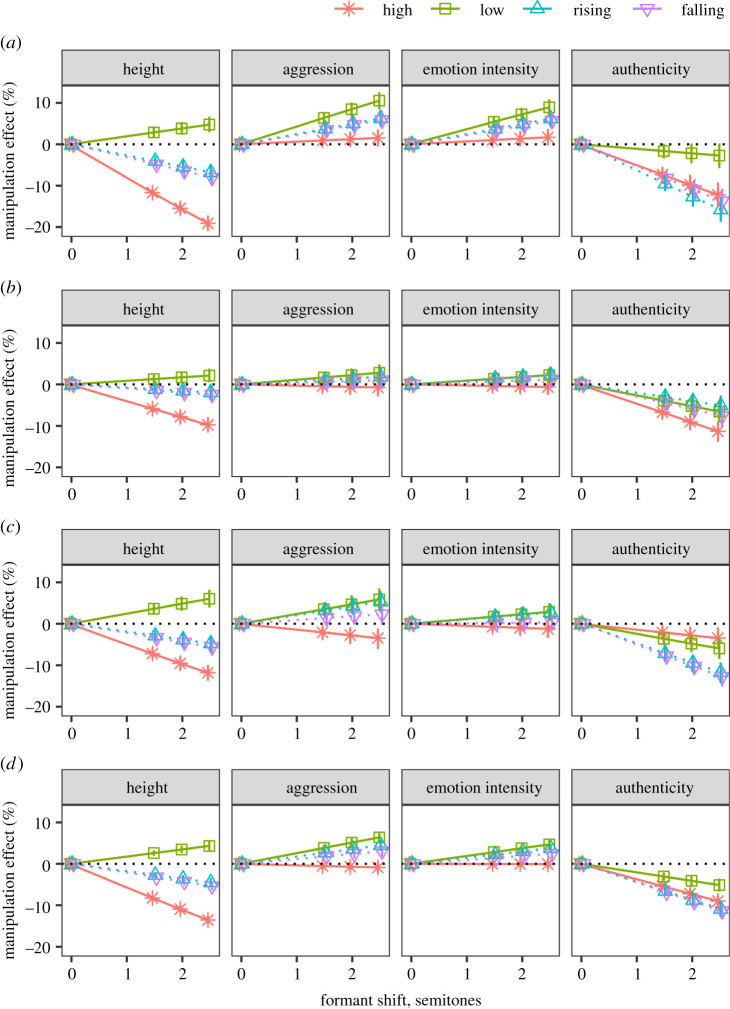
The effects of formant scaling for each manipulation strength (1.5, 2.0 or 2.5 semitones) relative to unmanipulated stimuli (0 semitones): medians of posterior distributions and 95% CIs. (*a*) English speech, (*b*) Persian speech, (*c*) synthetic roars and (*d*) average across English/Persian/roars.

Unaggregated responses from the tests of English speech, Persian speech and roars (47 993 trials) were pooled and analysed using a single Bayesian multilevel model fit with the R package *brms* [30]. Posterior distributions of model parameters and fitted values were summarized by their medians and 95% credible intervals (CIs). We compared credible values of effect sizes: when the credible intervals on estimates are far from the null value (e.g. zero), this indicates a credible effect given the observed data, model structure and prior knowledge. The outcome variable was the rating of a vocalization on a continuous scale (0 to 1), which was modelled with zero-one-inflated beta distribution [31]. The model predicted the rating in an individual trial as a function of Experiment (three levels: English speech, Persian speech and roars), Condition (four levels: low, high, rising and falling), Manipulation strength (four levels: 0, 1.5, 2 and 2.5 semitones) and Scale (four levels: height, aggression, emotion intensity and authenticity), with all possible interactions. Manipulation strength was treated as a continuous variable. Because a manipulation strength of zero means that there was no manipulation at all (i.e. control stimuli were identical in all four conditions), we set the corresponding beta coefficients to zero as part of prior specification; as a result, the regression lines were forced to converge at zero manipulation strength for each scale and experiment (figure 2). The effects of condition and scale were assumed to vary across subjects and across prototypes, and the effect of scale could also vary across individual stimuli. Finally, the variance of responses (phi) was assumed to vary across participants to account for individual differences in using the response scales. The model structure in *brms* syntax was as follows:

$$\begin{array}{rl} \mathrm{response} & ∼\mathrm{experiment}\times \mathrm{condition}\times \mathrm{manipulation}\_\mathrm{strength}\times \mathrm{scale}\\ & \;+(\mathrm{condition}\times \mathrm{scale}|\mathrm{subject}+\mathrm{prototype})+(\mathrm{scale}|\mathrm{stimulus}),\\ \mathrm{phi} & ∼(\mathrm{scale}|\mathrm{subject}). \end{array}$$

As a measure of inter-rater agreement in the rating task, we aggregated the ratings of each vocal stimulus on each response scale and calculated the mean Pearson's correlation between the responses of each participant and these aggregated ratings. These correlations ranged from *r* = 0.68 to 0.76 for height, aggression and emotion intensity scales, but were noticeably lower for the authenticity scale (*r* = 0.48). Likewise, the intraclass correlation coefficient, estimated using a two-way random model and absolute agreement, revealed lower reliability for the authenticity scale (0.14) compared to the other three response scales (0.30 to 0.43). This suggests that the perception of authenticity is more subjective and individually variable compared to speaker characteristics such as height and aggression.

The audio, datasets and R code for audio manipulation and data analysis are available in online supplements at http://cogsci.se/publications.html.

### 2.2. Results

From a single mixed model for all three stimulus types (English speech, Persian speech and roars), we calculated posterior distributions of contrasts between stimuli with shifted versus original formants, separately for each rating scale and experimental condition (statically high or low, dynamically rising or falling formants). These posterior distributions (figure 2) represent the most credible effect sizes for our manipulations, and they are presented here as percentage points (%), which in this case stands for the predicted change in fitted values of ratings on a scale of 0–100. The general pattern of findings was very similar for English speech, Persian speech and roars, the main difference being that effect sizes were smallest for Persian speech. A summary across all three stimulus types is available in figure 2*d*.

As expected, increasing the magnitude of formant manipulations amplified their perceptual effects. For example, the negative effect of statically *high* formants on perceived height in aggressive English speech increased from 11.7% (95% CI [10.5, 12.7]) to 19.1% [17.3, 20.7] as manipulation strength grew from 1.5 to 2.5 semitones. In the text, we present the effects for the strongest manipulation (2.5 semitones); complete results are available in figure 2.

Dynamic formant shifts (the *rising* and *falling* conditions) clearly affected the ratings on all four scales, but we observed no directional effects—no meaningful difference between the *falling* and *rising* conditions for any stimulus type or rating scale. Therefore, at least with this study design and the tested range of stimuli, it made no difference whether the speaker appeared to be gradually lengthening or shortening their vocal tract—only the amount of change in apparent VTL mattered. Instead, the effects of dynamic formant shifts on perceived height and aggression fell between those of statically high and statically low formants: the effect on perceived height was an attenuated version of the effect of statically high formants, while the effect on perceived aggression was an attenuated version of the effect of statically low formants (figure 2). This suggests that listeners based their judgement on a combination of the average apparent VTL and its extreme values; this basic finding is presented in more detail below.

#### 2.2.1. Height

In accordance with previous research, statically scaling all formant frequencies up (the *high* condition) made the speaker sound shorter by 19.1% [17.3, 20.7] in English speech, 9.7% [8.1, 11.3] in Persian speech and 11.8% [9.9, 13.8] in roars, while statically scaling formants down (the *low* condition) made the speaker sound taller by 4.8% [2.9, 6.7], 2.1% [0.5, 3.8] and 6.0% [3.9, 8.2] in English/Persian/roars, respectively, relative to unmanipulated stimuli. Notably, the effect sizes were considerably larger in the *high* versus *low* condition—that is, statically *high* formant frequencies made the speaker appear much shorter, while statically *low* formants increased perceived height only moderately.

Both dynamic manipulations had the effect of decreasing perceived height by approximately the same amount. When formants were gradually *rising* around the original average value, the speaker's perceived height decreased by 6.8% [4.8, 8.8], 1.9% [0.2, 3.6] and 4.8% [2.7, 6.8] in English/Persian/roars compared to unmanipulated stimuli with the same average formant frequencies. Likewise, gradually *falling* formants decreased perceived height by 8.1% [6.2, 9.9], 2.5% [0.8, 4.1] and 5.6% [3.7, 7.6] in English/Persian/roars, respectively.

In sum, statically high or low formants had the predicted effect on apparent height; however, contrary to predictions, formant scaling around their neutral values made the speaker appear shorter regardless of whether this corresponded to gradual vocal tract elongation or shortening.

#### 2.2.2. Aggression

Statically *high* formants had little or no effect on perceived aggression in English speech (1.5% [−0.6, 3.6]) and Persian speech (−0.7% [−2.9, 1.5]), and only a small negative effect in roars (−3.4% [−6.1, −1.1]). By contrast, statically *low* formants made the speaker sound considerably more aggressive in English speech (10.5% [8.4, 12.6]), and to a smaller extent also in Persian speech (2.8% [0.6, 4.9]) and in roars (5.9% [3.2, 8.6]). Dynamically *rising* and *falling* formants conveyed aggression in English (6.3% [4.2, 8.3] and 5.9% [3.7, 8], respectively) and in roars (5.3% [2.7, 7.9] and 2.3% [−0.3, 4.9], respectively), although in this case, the effect of *falling* formants was weaker and statistically uncertain. Thus, the effect of static formant manipulations on perceived aggression was in the predicted direction, but asymmetric (with lowered formants having a much more pronounced effect compared to raised formants), while dynamic formant shifts had the same perceptual effect regardless of their direction.

Curiously, most formant manipulations failed to change the aggression (and emotion intensity) ratings of Persian speech. Because formant manipulations in Persian speech did affect perceived speaker height, these changes must be perceptually salient, yet they were not interpreted by listeners as related to aggression levels, perhaps because the aggressive prosody in Persian was less pronounced or less familiar to the listeners.

#### 2.2.3. Emotion intensity

We observed very similar effects of formant manipulations on aggression and emotion intensity scales. Indeed, the responses on these two rating scales were strongly correlated (*r* = 0.72, while correlations between the remaining five pairs of scales were all negligible with *r* < 0.21). Because all stimuli conveyed different degrees of anger, it appears that participants interpreted the question about how aggressive the speaker was as largely synonymous with how emotional they were. Averaging across English/Persian/roars, perceived emotion intensity increased when formants were statically *low* (4.7% [3.6, 5.8]) or dynamically *rising* or *falling* (3.7% [2.7, 4.9] and 2.4% [1.3, 3.6], respectively), with no noticeable change when formants were statically *high* (−0.1% [−1.2, 1.1]).

#### 2.2.4. Authenticity

All stimuli were judged as relatively authentic, with a maximum loss in authenticity of around 10–15% at the strongest manipulation strength of 2.5 semitones compared to unmanipulated audio, without consistent differences among conditions. To some extent, however, any formant manipulation did make the utterance sound slightly less authentic relative to unmanipulated stimuli, as evidenced by the downward slope of all authenticity curves in figure 2. The likely reason is that the apparent VTL values in some stimuli may sometimes be pushed beyond the range consistent with the original pitch and voice quality: for example, if a male speaker already has an uncommonly short vocal tract, shortening it further creates an implausible heliox-like voice, in which the apparent female-range VTL is too short for the preserved male-range pitch. While this is to some extent unavoidable, we were primarily interested in whether static and dynamic formant manipulations had a similar effect on authenticity ratings. As it turned out, dynamic formant shift stimuli did not sound less natural than static formant scaling in speech. By contrast, dynamic formant shifts were judged to sound less authentic than static formant scaling in roars: by 3.5% [0.9, 5.9] and 6.0% [3.5, 8.5] in the *high* and *low* conditions, compared to 11.7% [9.3, 14.3] and 12.8% [10.3, 15.1] in the *rising* and *falling* conditions, respectively. It is worth reiterating that the authenticity scale had the lowest inter-rater reliability (see §2.1.5), and the perception of naturalness varied greatly across both listeners and stimuli.

## 3. Experiment 2

Our first experiment shows what happens when we gradually change the apparent VTL around its original value within a single aggressive utterance: the speaker sounds smaller and more emotional. While the design of Experiment 1 is good for studying perceptual biases, a more ecologically valid scenario is to model the acoustic consequences of dynamic vocal tract elongation or contraction from its neutral, relaxed position. Because the average formant frequencies are not preserved under such manipulations, we can no longer strictly distinguish between the effect of average VTL and its change over time. Instead, in Experiment 2, we test which strategy may be more effective for a caller trying to sound large and aggressive: to extend the vocal tract before starting to vocalize, or to let the audience hear the process of vocal tract elongation.

We simplified the design of Experiment 2 by using only the medium manipulation strength (2 semitones) and by focusing only on aggressive English speech, for which we had neutral recordings from the same speakers. A further methodological change in Experiment 2 was the addition of a baseline recording. Playing back isolated vocal stimuli represents a particular communicative context, namely hearing an aggressive utterance or call from a stranger whose ordinary voice quality is completely unknown. In that situation, a listener presumably has no way of knowing with certainty which level of shifting formant frequencies represents the natural VTL: for example, do rising formants correspond to a nervous smile or a return from fully depressed to neutral larynx position? To give the listeners the necessary background for interpreting VTL changes, in Experiment 2, we first presented listeners with a few seconds of neutral speech, followed by angry speech with manipulated or unmanipulated formants produced by the same speaker as the neutral speech. We predicted that the addition of a baseline would attenuate the effect of formant manipulations on apparent speaker size, while emphasizing the emotional significance of VTL changes. To take an extreme example, a sudden vocal tract elongation by a familiar individual, such as a family member whose body size is well known to the listener, is presumably interpreted solely in terms of emotions or intentions, not physical size.

### 3.1. Methods

#### 3.1.1. Stimuli

**Figure 3.**
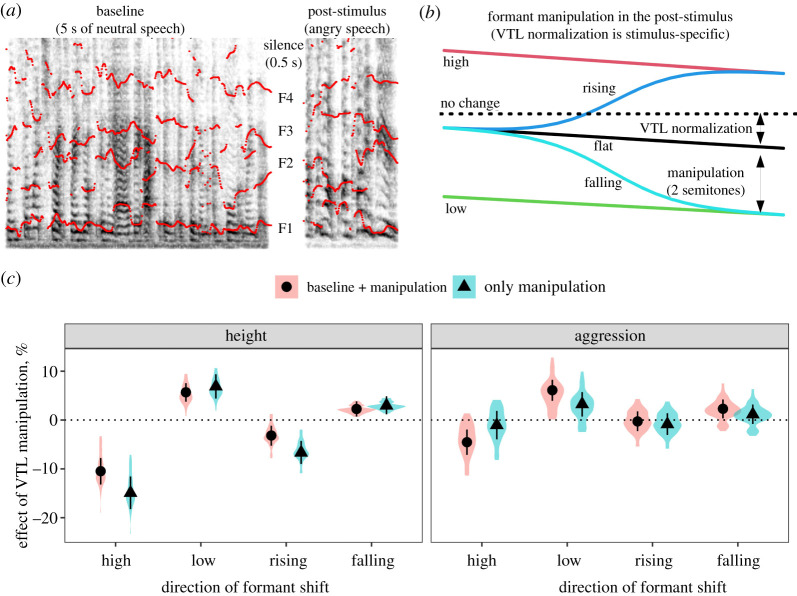
Design and results of Experiment 2. (*a*) An example of vocal stimulus. A baseline of a few seconds of neutral speech is followed by a post-stimulus—an aggressive utterance by the same speaker. (*b*) Post-stimulus manipulation: the initial apparent VTL is adjusted to match the average apparent VTL of baseline, and the general trend for changing apparent VTL in the post-stimulus is removed. In the example shown here, this results in lowering formant frequencies by about 0.4 semitones initially and 0.9 semitones towards the end of the post-stimulus. The ‘flattened’ post-stimulus is further manipulated to shift formant frequencies by ±2 semitones either at once (*high*/*low* conditions) or gradually, in an S-curve starting from the neutral value (*rising*/*falling* conditions). (*c*) The effect of dynamic formant manipulations in the post-stimulus compared to the flat condition: medians of posterior distributions and 95% CIs. Violin plots show the distribution of fitted values per prototype sound (*N* = 46).

Samples of aggressive English speech were mostly the same as in Experiment 1, but their number was increased from 39 to 46. Baseline recordings of neutral speech about 3–5 s in length were obtained from the same actors, who were asked to read the well-established neutral sentence ‘When sunlight strikes raindrops in the air, they act as a prism and form a rainbow’ [32]. All recordings were downsampled to 16 000 Hz and high-pass filtered over 50 Hz to prevent high- and low-frequency noise from introducing artefacts during resynthesis, then normalized for peak amplitude. Baseline recordings were not modified beyond this preprocessing. Post-stimuli (angry speech) had their formants shifted linearly without modifying the fundamental frequency, so as to match the VTL at baseline with the aggressive utterance and to remove the overall trend in apparent VTL in the post-stimulus. To achieve this normalization, we manually measured long-term average formant frequencies over the entire baseline, as well as separately over the first and second halves of the aggressive utterance, took the ratio of apparent VTLs and shifted formants dynamically by this linearly changing amount to flatten formant tracks in the post-stimulus (figure 3). After this preparation, formant frequencies in the post-stimuli were shifted by ±2 semitones statically or in a logistic curve either up (the *high* and *rising* conditions) or down (the *low* and *falling* conditions) relative to the *flat* condition. Therefore, there were 230 (46 prototypes × 5 conditions) unique post-stimuli, presented with or without a neutral baseline.

#### 3.1.2. Procedure

One sample of participants heard first a baseline recording (neutral speech), then after a 500 ms pause a post-stimulus (angry speech). Another independent sample of participants heard only the post-stimuli (angry speech), without a baseline. In both cases, the instructions were to rate the apparent speaker size or aggression expressed by the post-stimulus. Ratings on the size and aggression scales were given in two separate blocks of trials, and the order of both blocks and trials within block was chosen randomly for each participant. The vignettes and rating scales were the same as in Experiment 1.

#### 3.1.3. Participants

We recruited 332 participants (36% male, 63% female, 1% other/unspecified; age mean ± s.d. = 26 ± 7.4, range 18–56) on Prolific (https://app.prolific.co), who rated each stimulus on average 15.8 times on each of two rating scales.

#### 3.1.4. Data analysis

As in Experiment 1, we fitted a single Bayesian mixed model to all 17 596 trials:

$$\begin{array}{rl} \mathrm{response} & ∼\mathrm{experiment}\times \mathrm{condition}\times \mathrm{scale}\;\\ & \;+(\mathrm{experiment}\times \mathrm{condition}\times \mathrm{scale}|\mathrm{subject}+\mathrm{prototype})\\ & \;+(\mathrm{scale}|\mathrm{stimulus}),\\ \mathrm{phi}\; & ∼(1|\mathrm{subject}). \end{array}$$

Inter-rater reliability was similar to Experiment 1: mean correlation between a participant's ratings and the group average was *r* = 0.71 for height and *r* = 0.78 for aggression (intraclass correlation coefficient = 0.36 and 0.44, respectively).

### 3.2. Results

As predicted, overall, speakers sounded taller when formants were lowered, and shorter when formants were raised from the original level (figure 3*c*). However, these effects were much more pronounced if the formants were kept statically *high* or *low*, rather than gradually *rising* or *falling*. For example, without allowing listeners to hear the baseline speech of a vocalizer, the vocalizer was judged 14.9% (95% CI [11.6, 18.2]) shorter in the *high* formant condition versus 6.7% [4.3, 9.0] in the *rising* condition, and 6.9% [4.4, 9.4] taller in the *low* condition versus 3.0% [1.2, 4.9] in the *falling* condition. Interestingly, and in line with Experiment 1, *high* (or *rising*) formants had a much stronger effect on apparent body size compared to the effect of *low* (or *falling*) formants. In other words, high formants indicative of a short vocal tract were quite ‘costly’ in terms of greatly reducing perceived body size, whereas low formants achieved only a moderate amount of size exaggeration.

The addition of an unmanipulated baseline recording in a neutral voice noticeably attenuated the effect of formant manipulations on perceived height. For example, the effect of *rising* formants was reduced by half from 6.7% [4.3, 9.0] to 3.2% [1.2, 5.3] without a baseline (a difference of 3.5% [0.7, 6.3]). When judging body size, listeners were thus less ‘fooled’ by formant manipulations when they had access to the baseline VTL of the vocalizer in neural speech.

Formant manipulations had a weaker effect on perceived aggression than on perceived height. However, while the presence of a baseline attenuated the effect of formant manipulations on perceived height, it amplified their effect on perceived aggression. Without baseline speech, only statically *low* formants made the speaker sound slightly (3.2% [0.7, 5.7]) more aggressive. With a baseline, a speaker sounded more aggressive when formants were *low* (6.1% [3.9, 8.2]) or *falling* (2.3% [0.3, 4.2]), and less aggressive when they were *high* (4.5% [2.0, 7.1]), although we observed no effect of *rising* formants even with a baseline (0.3% [−2.3, 1.8]). When judging aggressive intent, listeners were thus more sensitive to formant manipulations when they were familiar with the speaker's natural voice.

## 4. General discussion

For a vocalizer caught up in a competitive interaction, sounding big is often the name of the game, whether it is a roaring contest of deer stags [14] or a TV debate of presidential candidates [33]. A popular vocal ‘trick’ is to extend the vocal tract and therefore lower its resonance frequencies or formants, projecting the impression of large size [3,4,7] and a dominant or aggressive attitude [12,18]. But what is the optimal strategy for acoustic size exaggeration: should the vocal tract be extended before or during the vocalization? More generally, how do listeners interpret audible VTL adjustments within a single aggressive nonverbal vocalization or speech utterance? To test this, we manipulated or synthesized a wide range of high-quality, ecologically valid stimuli, which included aggressive speech in a familiar and unfamiliar language, as well as fully controlled synthetic human nonverbal vocalizations. Our results show that, from the caller's perspective, fully extending the vocal tract before voice onset is more effective for acoustic size exaggeration, compared to letting the audience hear vocal tract extension. However, listeners do interpret gradual audible changes in apparent VTL as a sign of aggressive intent. We discuss these findings from an evolutionary perspective and tentatively identify the cognitive mechanisms involved.

From a vocalizer's perspective, our results show that static vocal tract elongation is more effective both for exaggerating body size and for sounding aggressive, at least in human vocalizations and speech. Scaling formant frequencies throughout an utterance has a much greater effect on perceived size and aggression than shifting them gradually to the same endpoints (Experiment 2). Moreover, when formant frequencies are shifted around some average value (Experiment 1), the resulting change in apparent VTL actually has a *negative* effect on apparent size compared to holding the VTL stable at the same average value, although it does make the caller sound slightly more emotional. For a vocalizer trying to intimidate, the safest bet is therefore to elongate their vocal tract before they start speaking or calling and to keep it extended, never letting the audience hear what the ‘short’ end of their vocal range sounds like.

It is important to emphasize that this finding applies specifically to aggressive vocal signals in humans and needs to be both replicated and extended. We focused on aggression because it maps most directly and uncontroversially on Ohala's frequency code [17]: if a particular vocal change makes a speaker sound larger or smaller, as VTL changes are known to do, it is highly likely to be relevant to the expression of aggression. In comparison, more ‘derived’ social characteristics, such as assertiveness or politeness, tend to be more contextually and culturally contingent [34]. Notably, our manipulations corresponded to both elongation and shortening of the vocal tract, but its shortening may arguably sound incongruous in aggressive contexts. This concern is mitigated by the finding that gradual VTL shortening was not perceived as less authentic than its gradual elongation in Experiment 1, but many questions remain for future studies. Crucially, dynamic VTL manipulations should also be investigated in affiliative or submissive contexts and vocalizations, building upon the recent work on auditory smiles [23].

Another important question is whether something akin to the experimentally created dynamic vocal tract scaling actually occurs in real life. Vocal gestures that affect VTL may simultaneously change its shape, in which case formant frequencies will not be scaled by the same amount. For example, smiling raises formant frequencies, while making a disgusted face lowers them, but in both cases lower formants, especially *F*1, are affected to a greater extent than upper formants [23,35]. Uniform formant scaling implemented in this study is therefore only an approximation of the acoustic consequences of various vocal gestures that affect VTL, which can in principle be modelled more precisely with an articulatory synthesizer [15]. With this proviso, rapid and perceptually salient changes in VTL are anatomically possible [12,13], and speakers who are asked to imitate a large or masculine person will increase their apparent VTL [36], sometimes by up to 4 cm [5]. The magnitude of VTL changes in our stimuli is thus realistic, but do VTL adjustments occur within a single utterance or vocalization? Our preliminary acoustic analysis of aggressive roars (electronic supplementary material) showed that in approximately one third of the analysed roars the vocal tract was scaled—most commonly shortened—by more than a semitone in the course of the vocalization, which should be detectable by listeners [4,7,27]. On the other hand, dynamic formant manipulations were judged to be less authentic than static manipulations in synthetic roars, whereas no such difference was apparent in speech stimuli. As for speech, vocal tract was clearly extended or shortened dynamically during many of the analysed aggressive English utterances, for which we attempted to correct when preparing the stimuli for Experiment 2. These changes are difficult to interpret, however, because speech articulation inevitably affects VTL. Furthermore, we do not know whether these VTL adjustments are communicative or incidental—for example, whether laryngeal depression in angry speech is simply a side effect of maximizing loudness when shouting or a way to counteract the tendency for voice pitch to rise with increasing vocal effort. In fact, classical singers are specifically trained to maintain a stable larynx to avoid a ‘tinny’ timbre even at the upper limit of their vocal range, whereas untrained singers tend to raise their larynx at high notes [13].

In other words, much more research is needed to establish how often, to what extent, and in what contexts dynamic vocal tract scaling is used communicatively in human vocal exchanges. Substantial VTL changes within a single vocalization are also observed in non-human animals. For example, red deer stags have movable larynges, which they pull down during roaring contests. A stag's roar typically begins with the larynx a little below its neutral position, and maximum laryngeal depression is achieved only toward the middle of the call, but in particularly intense harsh roars, the larynx is pulled down before the vocalization is given [37]. Based on our results, and from a functional perspective, this may be because roars produced with a *statically* low larynx sound more intimidating. A more general corollary is that audible changes in VTL within a single vocalization should be selected against in aggressive calls. It will be worthwhile to test these predictions and to document within-call VTL dynamics in aggressive and submissive calls of other species. It is also worth noting that we only tested a particular type of dynamic VTL change, namely a simple S-shape transition, while more complicated patterns comparable to pitch prosody may potentially occur. However, in comparison to nearly instantaneous pitch modulation over several octaves, VTL manipulation is rather slow (of the order of hundreds of milliseconds) and limited in scope (±20–25% at most, or a few semitones). It is therefore unlikely that the ‘language of VTL adjustments' is flexible enough to encode information in the precise shape of VTL contours, although this will need to be tested formally in future studies.

Moving from the signaller to the receiver, what perceptual and cognitive mechanisms might be involved in interpreting VTL dynamics? Judging by the results of Experiment 1, in which gradual shifts in formant frequencies preserved their average values, listeners used a combination of average and highest formants (shortest VTL) to estimate the height of vocalizers, and of average and lowest formants (longest VTL) to estimate their level of aggression. The general observation that audible changes in VTL affect perceived emotion, possibly because they are interpreted as an effort on the speaker's part to change voice quality, is in line with the results of Chuenwattanapranithi *et al*. [15]. Contrary to predictions [15,17], however, we found no evidence that the direction of change as such—vocal tract elongation or shortening—was informative; instead, the *rising* and *falling* conditions behaved very similarly in Experiment 1. The dynamic effect cannot be explained by the listeners attending preferentially to the initial or terminal VTL, as that would have created systematic differences between the *rising* and *falling* conditions, which we did not observe. Nor can a simple strategy of attending to the longest or shortest observed VTL account for the observed pattern of responses, because in that case, both dynamic conditions would align with the same static condition for all response scales. Instead, listeners appear to base their judgements partly on the average apparent VTL, but the range of implied VTL values introduces two opposite biases when judging size and aggression.

The results for perceived height are suggestive of what we might call a ‘disconfirmation bias'. In an aggressive context, it is in the speaker's interest to exaggerate their body size, but listeners are capable of both detecting attempted vocal deception of body size (about half the time) and adjusting their size estimates accordingly when deception is correctly detected [2]. When formants are experimentally lowered throughout an utterance, this presumably produces the impression of a genuinely long vocal tract. But an episode of elevated formant frequencies, however short, may be like Popper's black swan [38]—a single observation that suffices to falsify the hypothesis that the speaker is large. This would account for the negative effect of dynamically raising or lowering formants on perceived height in Experiment 1 and may explain why raising the formants relative to baseline in Experiment 2 caused the speaker to sound noticeably shorter, while lowering them had only a modest effect on apparent height. When it comes to physical size, listeners appear to expect some amount of vocal bluffing and bias their size estimates conservatively, towards the shortest implied VTL.

The opposite, namely a ‘confirmation bias’, was observed for aggression. In this case, the lowest observed formant values—longest VTL—biased aggression ratings in Experiment 1, and low or falling (rather than high or rising) formants had a much stronger effect on perceived aggression in Experiment 2. Thus, our data are generally in line with previous reports that the vocal tract is elongated in angry speech [12] and that lower formants convey aggression [18]. However, the effect appears to be independent of the direction of VTL change (contrary to [15])—a drop in VTL can occur at the beginning or end of an utterance and still be perceived as aggressive. Furthermore, the effect sizes for aggression were modest compared to the large impact of formant scaling on perceived size. Interestingly, the effect of VTL changes on aggression was amplified when the aggressive stimuli were preceded by a neutral baseline. Including a baseline familiarizes listeners with each speaker's normal, relaxed voice; unsurprisingly, dynamic formant shifts become less informative of height when preceded by such a baseline. By the same token, however, having a baseline makes it easier to notice when the VTL implied by shifted formants goes beyond the speaker's habitual range, potentially increasing the emotional significance of these changes.

Interestingly, the perceptual effects of all VTL manipulations were considerably more pronounced in a familiar language (English) compared to an unfamiliar one (Persian), which is consistent with the hypothesis that the separation of phonemic and size-related processing of formant patterns is not an encapsulated low-level auditory process, but can be modulated by contextual and semantic cues [39]. In addition, all English speech fragments were repetitions of the same phrase, and this predictability may have further facilitated the perceptual task of tracking VTL dynamics. The synthetic roars were also predictable in the sense that their formant tracks were always perfectly parallel, which is not very realistic anatomically, but makes it straightforward for listeners to detect VTL changes. Indeed, the magnitude of perceptual effects of formant shifts in roars was comparable to that in English speech. Therefore, it appears that the estimation of speaker size from vocal tract length is more effective when there are few concurrent articulatory changes (as in roars), or when top-down expectations can facilitate the process of vowel normalization (as when listening to a familiar language).

Summing up, the take-home message for vocalizers trying to sound genuinely large and intimidating using formant modulations is to keep their vocal tract extended *consistently*. However, VTL perception in humans appears to be quite sophisticated, involving a variety of perceptual biases, familiarity effects, task-dependent asymmetric weighting of apparent vocal tract elongation and contraction, and quite likely other factors that we were unable to address directly in the current study, such as cross-modal associations with facial expressions (e.g. auditory smile, lip protrusion, gaping), physiological motor acts (choking) and socio-cultural factors. Accordingly, a range of meanings can potentially be expressed with dynamic VTL adjustments. How these complex aspects of vocal production and perception relate to the strategies used in real-life vocal interactions in humans and other species, as well as to the evolution of vocal communication, is a subject for future research.

## Data accessibility

All datasets, R scripts for data analysis, audio stimuli, R scripts for generating synthesized stimuli and html code for running psychoacoustic experiments are available as electronic supplementary material, or can be downloaded from http://cogsci.se/publications.html. These supplemental materials enable full validation and replication of results. The data are provided in the electronic supplementary material [40].
